# Bioseparation of Four Proteins from *Euphorbia characias* Latex: Amine Oxidase, Peroxidase, Nucleotide Pyrophosphatase/Phosphodiesterase, and Purple Acid Phosphatase

**DOI:** 10.1155/2011/369484

**Published:** 2011-10-15

**Authors:** Rosaria Medda, Francesca Pintus, Delia Spanò, Giovanni Floris

**Affiliations:** Dipartimento di Scienze della Vita e dell'Ambiente, Università di Cagliari, Cittadella Universitaria, 09042 Monserrato, Italy

## Abstract

This paper deals with the purification of four proteins from *Euphorbia characias* latex, a copper amine oxidase, a nucleotide pyrophosphatase/phosphodiesterase, a peroxidase, and a purple acid phosphatase. These proteins, very different in molecular weight, in primary structure, and in the catalyzed reaction, are purified using identical preliminary steps of purification and by chromatographic methods. In particular, the DEAE-cellulose chromatography is used as a useful purification step for all the four enzymes. The purification methods here reported allow to obtain a high purification of all the four proteins with a good yield. This paper will give some thorough suggestions for researchers busy in separation of macromolecules from different sources.

## 1. Introduction

**Figure 1:**
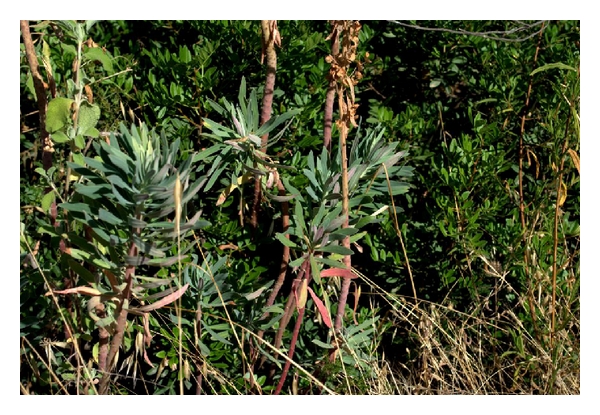
*Euphorbia characias*, the Mediterranean spurge.

*Euphorbia characias *(Figure 1) is a shrub belonging to the Euphorbiaceae family commonly occurring in various habitats—rocky hillsides, along road verges, in open woods, and in olive groves—in vast areas of the Mediterranean basin. Characteristic of all Euphorbiaceae is the presence of laticifers [1, 2], specialized cells forming vessel-like structures containing latex, a milky sap with a complex composition, which includes alkaloids, terpenoid compounds, and a number of enzymes [3]. Previous studies have shown the presence of several proteins in the latex of *Euphorbia characias*, some of these had been purified and characterized.

### 1.1. Amine Oxidase

(amine: oxygen oxidoreductase (deaminating; copper containing); EC 1.4.3.6.) One of these proteins is an amine oxidase [4] (*Euphorbia* latex amine oxidase; ELAO), a homodimeric protein, each subunit (molecular mass ≅ 74 kDa) containing an active site composed by a tightly bound Cu^2+^ and a quinone of 2,4,5-trihydroxyphenylalanine (TPQ or TOPA) [5]. TPQ is derived from the post-translational copper-catalyzed oxidation of a modified tyrosine residue in the consensus sequence Asn-Tyr-Asp-Asn of the polypeptide chain (GenBank accession number AF171698) [6]. The copper ion is coordinated with the imidazole groups of three conserved histidine residues and with two water molecules (equatorial We and axial Wa). TPQ, close but not bound to the Cu^2+^, appears to have high rotational mobility. Owing to the presence of the TPQ cofactor, the oxidized form of ELAO has a distinctive pink colour and absorbs, in the visible region, at 490 nm (*ε*_490_ = 6000 M^−1^ cm^−1^) [4]. ELAO oxidizes the primary amines with the formation of the corresponding aldehyde, ammonia, and hydrogen peroxide. The ping-pong catalytic mechanism of ELAO, similar to all copper amine oxidases, can be divided into two half reactions: the first, referred to as “reductive half reaction,” involves the oxidation of amine to aldehyde and the formation of a reduced form of TPQ cofactor:

(1) $${\text{E}}_{\text{ox}}+\text{R–C}{\text{H}}_{2}\text{–N}{\text{H}}_{3}^{+}\to {\text{E}}_{\text{red}}+\text{R–CHO}.$$

The second half reaction, known as “oxidative half–reaction”, involves the reoxidation of the enzyme with the contemporarily release of ammonia and hydrogen peroxide:

(2) $${\text{E}}_{\text{red}}+{\text{O}}_{2}+{\text{H}}_{2}\text{O}\to {\text{E}}_{\text{ox}}+\text{N}{\text{H}}_{4}^{+}+{\text{H}}_{2}{\text{O}}_{2}.$$

### 1.2. Heme-Containing Peroxidase

(EC 1.11.1.7; donor: hydrogen peroxide oxidoreductase). Another well-characterized protein from *Euphorbia characias* latex is a cationic peroxidase (*Euphorbia* latex peroxidase: ELP) [7], belonging to a “non animal” class III secreted plant peroxidases, well-known enzymes oxidizing a variety of aromatic molecules in the presence of hydrogen peroxide [8]. ELP is formed by a single glycosylated polypeptide chain of 347 residues with a relative molecular mass of 47 KDa and contains a ferric iron-protoporphyrin IX in a quantum mechanically mixed-spin state, pentacoordinated to a “proximal” histidine ligand. The ELP sequence (GenBank accession number AY586601) permits to identify two highly conserved histidine residues coordinated to the heme (His_50_ and His_179_ distal and proximal, respectively). Alike other secreted plant peroxidases, ELP has two calcium-binding sites, namely “proximal” and “distal,” but the purified protein contains only one mole of endogenous Ca^2+^/mol enzyme strongly bound to the proximal site [7]. This calcium ion plays a critical role in retaining the active site geometry of the enzyme. The addition of a second Ca^2+^ ion to the distal site of the native ELP is necessary in order to express the full activity of the enzyme.

### 1.3. Nucleotide Pyrophosphatase/Phosphodiesterase

(EC 3.1.4.x; EC 3.6.1.x). Nucleotide pyrophosphatase/phosphodiesterases (NPPs), members of the alkaline phosphatase superfamily, represent a group of widely distributed proteins releasing nucleoside 5′-monophosphates from nucleotides and their derivates.

Several NPPs from plants have been characterized [9]. These glycosylated proteins occur in membrane systems, accumulate in vacuoles, or are secreted from the cells [10]. Plant NPPs seem to play a crucial role specially in diverting carbon flux from starch and cell wall polysaccharide biosynthesis to other metabolic pathways [11].

A soluble nucleotide pyrophosphatase/phosphodiesterase, purified to homogeneity from *Euphorbia characias* latex (ELNPP) [12], has a molecular mass of 80 ± 5 kDa and is formed by two apparently identical subunits, each containing one Ca^2+^ and one Mg^2+^ ion. ELNPP exhibits hydrolytic activities toward pyrophosphate/phosphodiester bonds of a broad range of substrates and very efficiently hydrolyzes the artificial substrate thymidine 5′-monophosphate 4-nitrophenyl ester generating 4-nitrophenolate as a final product.

### 1.4. Purple Acid Phosphatase

Phosphatases are enzymes usually classified as acid or alkaline depending on their pH optimum, for catalytic activity, below or above pH 7.0. Acid phosphatases (APs; EC 3.1.3.2) are ubiquitous enzymes ranging from microorganisms (fungi and bacteria), plants, and animals [13, 14]. APs catalyze the hydrolysis of phosphate (Pi) from phosphate monoesters, and their function seems to be involved in the release, transport, and recycling of Pi, a crucial macronutrient for cellular metabolism and bioenergetics.

Purple acid phosphatases (PAPs) represent a distinct class of acid phosphatases. Their typical absorbance spectrum at about 530–560 nm, with a characteristic purple color, is due to a charge-transfer transition between a tyrosine residue and a coordinated ferric iron [15]. These enzymes contain a metal center Fe(III)-Me(II), where Me(II) can be Zn, Mn, or Fe. Plants contain two major groups of PAPs: small PAPs, 35–40 kDa monomeric proteins homologous to mammalian enzymes, and large PAPs, 110–130 kDa homodimeric proteins, with or without a disulfide bridge between the two subunits [16].

A purple acid phosphatase, purified to homogeneity from *Euphorbia characias* latex (ELPAP) [17], has a molecular mass of 130 ± 10 kDa and is formed by two apparently identical subunits each containing one Fe(III) and one Zn(II) ion. The two subunits are connected by a disulfide bridge. The enzyme has an absorbance maximum, in the visible region, at 540 nm which confer a characteristic purple color, and the tyrosine residue coordinated to the ferric iron is Tyr_172_ (GenBank accession number HM641814).

## 2. Purification

The four enzymes above reported are purified by the following steps.

*Plant*: Four hundred mL *Euphorbia characias* latex, drawn from cut branches, is collected at several locations in southern Sardinia (Italy), immediately frozen at −80°C, lyophilized, and stored at −20°C until use.

**Step 1**

*Acetone powder.* The lyophilized is poured into 1 L of cold acetone and kept for 45 min with constant stirring at −20°C. The acetone powder is collected by filtration on a Buchner's funnel. Acetone powder (30 g) is mixed with 700 mL H_2_O with continuous stirring for 120 min and centrifuged at 9000 rpm for 45 min and the precipitate discarded.

**Step 2 (for ELAO, ELP, and ELPAP)**

*Ammonium sulfate fractionation*. The supernatant is made 25% saturated with ammonium sulfate with constant stirring for 30 min and centrifuged at 9000 rpm for 30 min. The precipitate is discarded, and the supernatant is brought to 80% saturation with ammonium sulfate with constant stirring for 30 min and centrifuged at 9000 rpm for 30 min. The pellet is dissolved in:

(a) 40 mL of 10 mM K^+^-phosphate buffer, pH 7.0, and dialyzed for 12 h against the same buffer for the purification of ELAO and ELP,

(b) 40 mL of 10 mM Tris/HCl buffer, pH 7.0, and dialyzed for 12 h against the same buffer for ELPAP purification.

**Step 2 (for ELNPP)**

*Controlled heating at *60*°*C. The supernatant (Step 1) is brought to 60°C and kept at this temperature for 30 min in a thermostatic water bath under continuous stirring. After rapid cooling in ice water, the suspension is centrifuged at 9000 rpm for 30 min and the precipitate discarded.

**Step 3 (for ELAO and ELP)**

*DEAE-cellulose chromatography.* The dialyzed (Step 2(a)) is loaded onto a DEAE-cellulose column (2.8 × 14 cm) equilibrated and washed with 10 mM K^+^-phosphate buffer, pH 7.0. In these conditions, ELP is not bound to the resin and is collected until the A_280_ became ≤ 0.01. The column is washed with 100 mM K^+^-phosphate buffer, pH 7.0. ELAO is then eluted with 125 mM K^+^-phosphate buffer, pH 7.0. The fractions with the same specific activity are pooled and dialyzed against 10 mM K^+^-phosphate buffer, pH 7.0.

**Step 3 (for ELNPP)**

*Ammonium sulfate fractionation.* This step is identical to Step 2(a) for ELAO and ELP.

**Step 3 (for ELPAP)**

*DEAE-cellulose chromatography*. The dialyzed (Step 2(b)) is loaded onto a DEAE-cellulose column (2.8 × 14 cm) equilibrated and washed with 10 mM Tris/HCl buffer, pH 7.0. The enzyme is then eluted with 100 mM Tris/HCl buffer, pH 7.0. The fractions with the same specific activity are pooled, dialyzed for 12 h against 100 mM Tris/HCl buffer, pH 7.5, containing 300 mM NaCl, and concentrated by filtration under vacuum.

**Step 4 (for ELAO and ELP)**

*Hydroxylapatite column chromatography*. The eluates from Step 3 are independently loaded onto a hydroxylapatite column (2.8 × 10 cm) equilibrated and washed with 10 mM K^+^-phosphate buffer, pH 7.0. Afterwards the column is washed with the following.

- 25 mM K^+^-phosphate buffer, pH 7.0, and ELAO is eluted with 50 mM K^+^-phosphate buffer, pH 7.0. The fractions with the same specific activity are pooled.
- 200 mM K^+^-phosphate buffer, pH 7.0, and ELP is eluted with 400 mM K^+^-phosphate buffer, pH 7.0. The fractions with the same specific activity are pooled and concentrated by filtration under vacuum. Homogeneous ELP is obtained, and Table 1 presents the purification method for this enzyme which is purified 270-fold with a yield of about 40%.

**Step 4 (for ELNPP)**

*DEAE-cellulose chromatography*. The dialyzed (Step 3) is loaded onto a column (2.8 × 14 cm) equilibrated and washed with 10 mM K^+^-phosphate buffer, pH 7.0. In these conditions, the enzyme is not bound to the resin and is eluted and collected until the A_280_ became ≤ 0.01. The fractions with the same specific activity are pooled and concentrated by filtration under vacuum. Homogeneous ELNPP is obtained. The purification procedure of ELNPP is reported on Table 2. The enzyme is purified 26-fold with a yield of about 40%.

**Step 4 (for ELPAP)**

*Gel filtration chromatography*. The concentrated solution from Step  3 is loaded onto a column (2 × 100 cm) of Sephacryl S-200 (fine grade) equilibrated and eluted at 4°C with 100 mM Tris/HCl buffer, pH 7.5, containing 300 mM NaCl. The fractions with the highest specific activity are pooled and concentrated by filtration under vacuum. Homogeneous ELPAP is obtained, and Table 3 presents a summary of the purification procedure for ELPAP. The enzyme is purified 83-fold with a yield of about 14%.

**Step 5 (for ELAO)**

*AH-Sepharose column chromatography*. The eluate from Step 4(b) is loaded onto an AH Sepharose column (2.0 × 10 cm) equilibrated and washed with 50 mM K^+^-phosphate buffer, pH 7.0. The enzyme is eluted with 100 mM K^+^-phosphate buffer, pH 7.0. The fractions with the same specific activity are pooled and concentrated by filtration under vacuum. Homogeneous ELAO is obtained.  Table 4 presents a summary of the purification procedure for ELAO. The enzyme was purified 52-fold with a yield of about 61%.

**Scheme 1:**
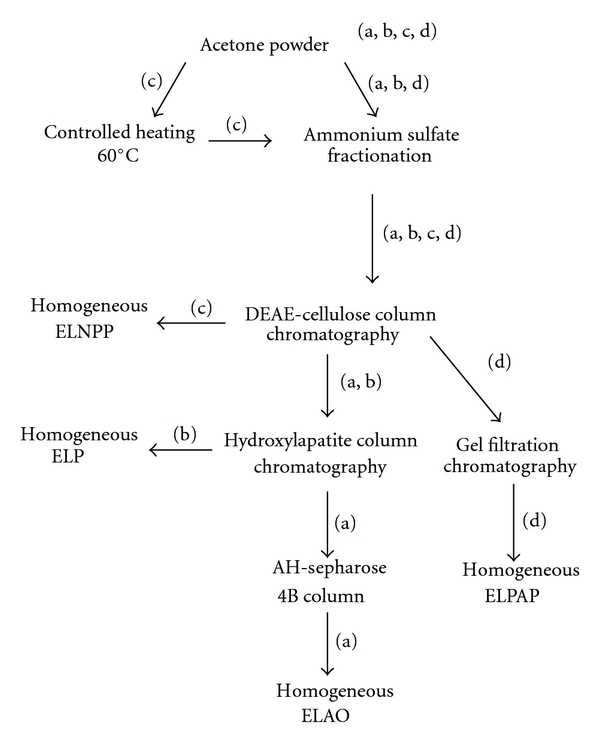
Steps  of the purification methods for the enzymes from *Euphorbia* latex. (a) ELAO; (b) ELP; (c) ELNPP; (d) ELPAP.

Scheme 1 represents a summary of the purification procedure utilized for all the four proteins.

### 2.1. Affinity Chromatography: An Alternative Method for Purification of ELP

The amino acid sequence of ELP reveals the presence of a putative CaM-binding domain between residues 26–39 of ELP, a 14 aa sequence (IQKELKKLFKKDVE) with the characteristics of a IQ-like motif. In addition, a related motif for CaM binding, termed 1–8–14, is spotted between residues 79–92 (LSLRKQAFKIVNDL; GenBank accession number AY586601) [18]. Thus, CaM immobilized on Sepharose 4B (CaM-Sepharose) is used as an affinity chromatography in the purification of *Euphorbia *peroxidase [19]. 

ELP samples from Step 2 are dialyzed against buffer A (50 mM Tris/HCl, pH 7.4, 5 mM CaCl_2_) and applied on a CaM-sepharose column (2.5 × 6.5 cm) equilibrated with buffer A at flow rate of 10 mL/h. The column is washed with the same buffer until the optical density at 280 nm of the effluent became about 0.001. ELP is strongly bound to the column. After washing the column with buffer B composed as A but containing 1 M NaCl in order to remove nonspecifically bound proteins, ELP is eluted from the column with 25 mM Tris/HCl buffer, pH 7.4, containing 2 mM EGTA, a calcium-chelating agent, and then dialyzed in 100 mM K^+^-phosphate buffer, pH 7.0. In the absence of calcium ions in the buffer A, ELP is not able to bind to the column and is immediately eluted. The scheme of the purification procedure is shown in Table 5. The recovered total activity is higher to that obtained with the previously described method [20]. 

### 2.2. Polyacrylamide Gel Electrophoresis

**Figure 2:**
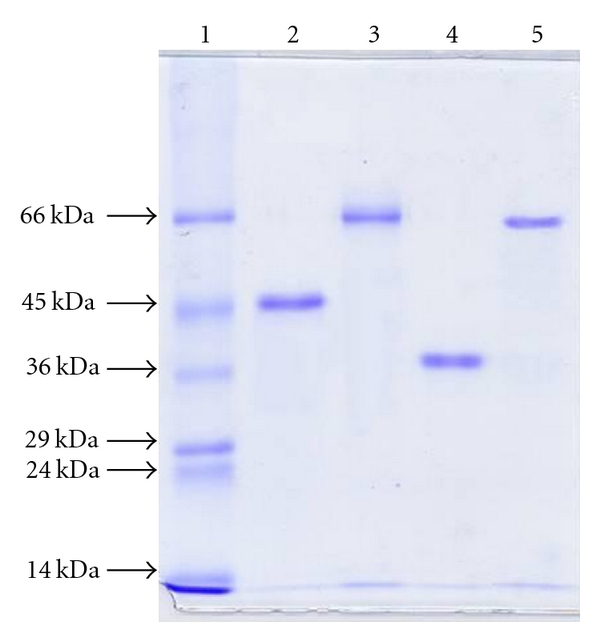
SDS-PAGE. Protein samples after the purification steps. Line 1: standard molecular weights: bovine serum albumin (66 kDa), ovalbumin (45 kDa), glyceraldehydes-3-phosphate dehydrogenase (36 kDa), carbonic anhydrase (29 kDa), trypsinogen (24 kDa), and *α*-lactalbumin (14.2 kDa); line 2: ELP; line 3: ELAO; line 4: ELNPP; line 5: ELPAP.

For all the proteins, one protein band with enzymatic activity is present on PAGE in nondenaturing conditions (not shown), and one protein band is observed on SDS-PAGE (Figure 2).

### 2.3. Spectroscopic Features

**Figure 3:**
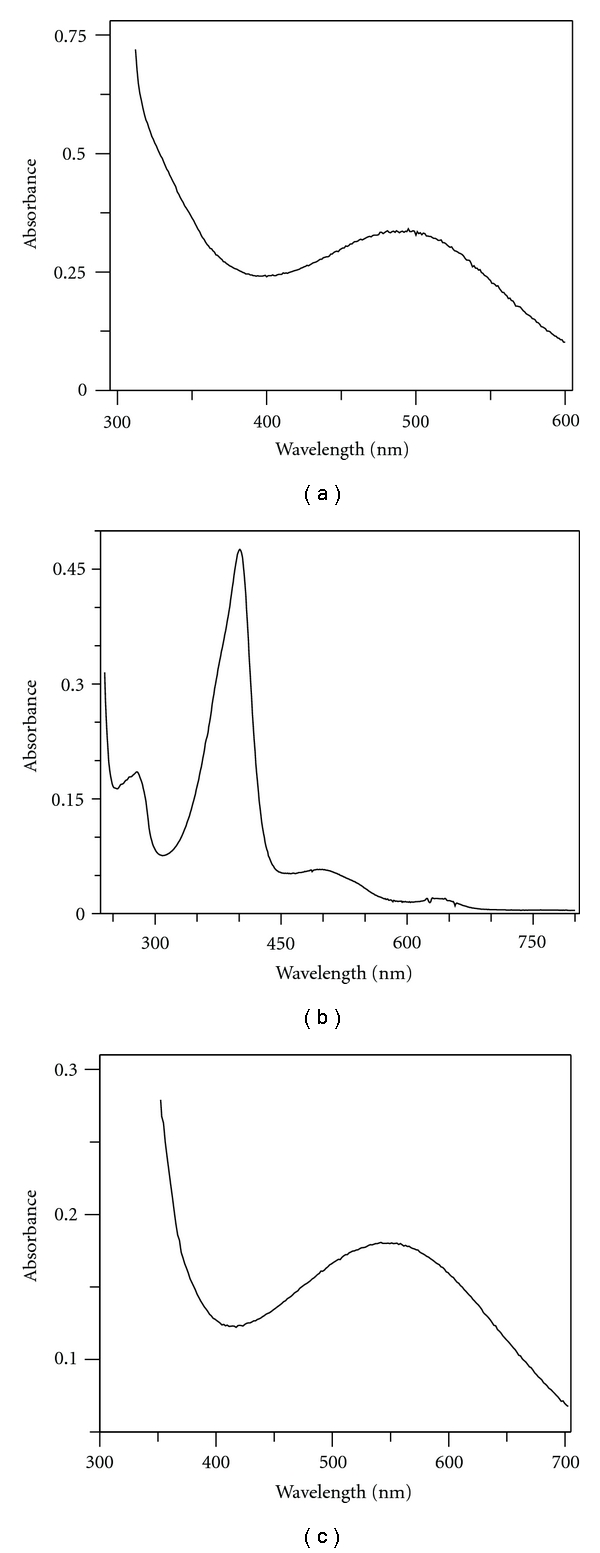
Visible absorption spectrum of homogeneous (a) *Euphorbia *amine oxidase (56 *μ*M), (b) *Euphorbia* peroxidase (3.6 *μ*M), and (c) *Euphorbia* purple acid phosphatase (62 *μ*M). Buffer used: ELAO and ELP 100 mM K^+^-phosphate, pH 7.0; ELPAP 100 mM Tris/HCl, pH 7.0.

Figure 3 shows the visible absorption spectrum of homogeneous ELAO (a), ELP (b), and ELPAP (c). For ELP, we obtain an Reinheitzahl (RZ) value A_401_/A_278_ = 2.7, typical of highly purified peroxidase.

## 3. Conclusions

The wide distribution in all living organisms of all the four enzymes above reported suggests that they could be of great biological importance, but the physiological functions and the metabolic control of these enzymes are still poorly understood. There is evidence of the coexistence of multiple enzymatic activities within the latex-driving system of *Euphorbia characias*. For instance, an amine oxidase, a copper/quinone-containing enzyme, catalyzing an oxidative deamination, controls the level of mono-, di- and polyamines and, presumably, participates, through the production of hydrogen peroxide, to the defensive oxidative burst. Scavenging of H_2_O_2_ is mediated by the Ca^2+^/CaM-regulated peroxidase. *Euphorbia* peroxidase utilizes hydrogen peroxide to oxidize a second reducing substrate and, by its hydroxylic cycle, forming ROS, presumably participates to the defensive oxidative burst [7].

The pyrophosphatase/phosphodiesterase and the purple acid phosphatase in *Euphorbia* seem, again, to be related in the plant metabolism. ELNPP can produce phosphomonoesters from diesters of phosphoric acid, and these monoesters can be well hydrolyzed by ELPAP taking to hypothesize an joined action of these important enzymes on the metabolism of nucleotides [17]. Moreover, the ELPAP activity could ensure an adequate Pi supplement in *Euphorbia* shrub.

It is worth to note that the purification method of all the enzymatic proteins reported here allows to obtain homogeneous highly purified enzyme samples with good yield. In particular, a recovery of 40% and 61% for ELP and ELAO, respectively, is obtained. Moreover, the mild stepwise elution is used varying the buffer ionic strength without any elution gradient making fast and easy reproducible methods. This paper will give some thorough suggestions for researchers busy in separation of macromolecules from different sources in order to study *in vitro* reconstituted coupled enzymatic systems in all the forms of life.

## Figures and Tables

**Table 1:** Purification of *Euphorbia* latex peroxidase.

Step	Total protein (mg)	Total activity (nkat)	Specific activity (nkat/mg)	Yield (%)	Purification (fold)
Acetone powder crude extract	3500	71545	20.4	100	1
Ammonium sulfate fractionation	534	40910	76.6	57	3.7
DEAE-cellulose chromatography	20.3	33414	1646	46.7	80.7
Hydroxylapatite column chromatography	5.2	28455	5500	39.8	270

**Table 2:** Purification of *Euphorbia* latex nucleotide pyrophosphatase/phosphodiesterase.

Step	Total protein (mg)	Total activity (nkat)	Specific activity (nkat/mg)	Yield (%)	Purification (fold)
Acetone powder crude extract	3500	9500	2.7	100	1
Controlled heating (60°C)	360	6665	18.5	70	6.8
Ammonium sulfate fractionation	210	5615	26.7	59	10
DEAE-cellulose chromatography	53	3780	71.3	40	26

**Table 3:** Purification of *Euphorbia* latex purple acid phosphatase.

Step	Total protein (mg)	Total activity (nkat)	Specific activity (nkat/mg)	Yield (%)	Purification (fold)
Acetone powder crude extract	3500	418567	119.6	100	1
Ammonium sulfate fractionation	534	165133	309	39.4	2.6
DEAE–cellulose chromatography	101	92700	917.8	22	7.6
Gel filtration chromatography	5.9	58567	9927	14	83

**Table 4:** Purification of *Euphorbia* latex copper amine oxidase.

Step	Total protein (mg)	Total activity (nkat)	Specific activity (nkat/mg)	Yield (%)	Purification (fold)
Acetone powder crude extract	3500	37917	10.8	100	1
Ammonium sulfate fractionation	534	33817	63	89.2	5.8
DEAE-cellulose chromatography	200.3	30000	150	79	13.8
Hydroxylapatite column chromatography	80.2	28067	350	74	32
*ω*-Aminohexy-Sepharose 4B column chromatography	41	23233	567	61	52.5

**Table 5:** Purification of *Euphorbia* latex peroxidase by affinity chromatography.

Step	Total protein (mg)	Total activity (nkat)	Specific activity (nkat/mg)	Yield (%)	Purification (fold)
Acetone powder crude extract	3500	71545	20.4	100	1
Ammonium sulfate fractionation	534	40910	76.6	57	3.7
CaM-sepharose column chromatography	5.9	33536	5700	46.8	279
